# Effect of Zirconium Oxide and Zinc Oxide Nanoparticles on Physicochemical Properties and Antibiofilm Activity of a Calcium Silicate-Based Material

**DOI:** 10.1155/2014/975213

**Published:** 2014-11-06

**Authors:** Juliane Maria Guerreiro-Tanomaru, Adinael Trindade-Junior, Bernardo Cesar Costa, Guilherme Ferreira da Silva, Leonardo Drullis Cifali, Maria Inês Basso Bernardi, Mario Tanomaru-Filho

**Affiliations:** ^1^Department of Restorative Dentistry, Araraquara Dental School, São Paulo State University (UNESP), Rua Humaitá 1680, P.O. Box 331, 14.801-903 Araraquara, SP, Brazil; ^2^Institute of Physics, University of São Paulo (USP), Avenida Trabalhador São-Carlense 400, 13566-590 São Carlos, SP, Brazil

## Abstract

The aim of the present study was to evaluate the antibiofilm activity against *Enterococcus faecalis*, compressive strength. and radiopacity of Portland cement (PC) added to zirconium oxide (ZrO_2_), as radiopacifier, with or without nanoparticulated zinc oxide (ZnO). The following experimental materials were evaluated: PC, PC + ZrO_2_, PC + ZrO_2_ + ZnO (5%), and PC + ZrO_2_ + ZnO (10%). Antibiofilm activity was analyzed by using direct contact test (DCT) on *Enterococcus faecalis* biofilm, for 5 h or 15 h. The analysis was conducted by using the number of colony-forming units (CFU/mL). The compressive strength was performed in a mechanical testing machine. For the radiopacity tests, the specimens were radiographed together with an aluminium stepwedge. The results were submitted to ANOVA and Tukey tests, with level of significance at 5%. The results showed that all materials presented similar antibiofilm activity (*P* > 0.05). The addition of nanoparticulated ZnO decreased the compressive strength of PC. All materials presented higher radiopacity than pure PC. It can be concluded that the addition of ZrO_2_ and ZnO does not interfere with the antibiofilm activity and provides radiopacity to Portland cement. However, the presence of ZnO (5% or 10%) significantly decreased the compressive strength of the materials.

## 1. Introduction

Calcium silicate- (CS-) based materials, such as mineral trioxide aggregate (MTA), have been widely used in dentistry for different applications, including pulp capping and pulpotomy, sealing of radicular or furcation perforations, internal or external resorption, apexification, and root-end filling in endodontic surgery due to its excellent biological and adequate physicochemical properties [1–4].

Portland cement (PC) is the main component of MTA [1, 5] and both show similar physicochemical and biological characteristics [6, 7]. It has been reported that Portland cement exhibits biocompatibility and high compressive strength allowing this material to be also suitable for medical indications, such as in orthopedic applications [8]. However, PC does not exhibit the radiopacity required to differentiate from surrounding anatomic structures. So MTA composition includes bismuth oxide (Bi_2_O_3_) as radiopacifier agent [3, 9–11].

Despite advantages of MTA when compared to other materials, it is well documented that Bi_2_O_3_ interferes with some properties of the material. Bismuth oxide interferes with the hydration mechanism of MTA and precipitation of calcium hydroxide in the hydrated paste [12]. Furthermore, the association of Bi_2_O_3_ changes the microstructure of the cement by acting as flaws within the cement matrix [13] and, consequently, increases the porosity and solubility of the Portland cement reducing its resistance [13, 14]. Regarding the biocompatibility, it has been shown that Bi_2_O_3_ interferes with cell growth [15], increases the cytotoxicity of the material [16], and promotes inflammatory reaction on subcutaneous tissue [14].

Thus, the association of PC with other radiopacifier agents has been studied [10, 11, 17–19]. Among several radiopacifiers studied in association with PC, zirconium oxide (ZrO_2_) has demonstrated satisfactory results [18, 19]. The association of PC with 30% of ZrO_2_ exhibits radiopacity, compressive strength, setting time, water absorption, and solubility similar to MTA ProRoot and resulted in a calcium silicate hydrated calcium hydroxide and a minimum amount of monosulfate and ettringite (natural hydrous calcium aluminum sulfate) [17]. It was already observed that the association of PC with ZrO_2_ promoted satisfactory pH, solubility, calcium ions release, and setting time [14]. It was also verified that zirconium oxide added to Portland cement promoted better biological response than association of bismuth oxide to PC in rat subcutaneous tissue [14].

The addition of nanoparticulated substances to different materials can improve some properties, such as the antimicrobial activity. Zinc oxide (ZnO) is currently being investigated as an antibacterial agent in both microscale and nanoscale formulations. Results have indicated that ZnO nanoparticles show antibacterial activity greater than for microparticles [20]. Besides, it was already demonstrated that the use of nanoparticulated zinc oxide (ZnO) increases the antimicrobial activity of some products, including against biofilms of* Staphylococcus aureus* and* Enterococcus faecalis* [21–24].

The antimicrobial potential of MTA and PC has been evaluated by using agar diffusion test, showing that PC-based cements have antimicrobial activity against some microorganisms [25]. Additionally to agar diffusion test, the antibiofilm activity can be performed by direct contact test (DCT) on* Enterococcus faecalis* biofilm as proposed by Faria-Júnior et al. [26]. When evaluated by this method, the Sealapex and MTA Fillapex sealers promoted reduction of bacteria on biofilms. The authors concluded that the direct contact test is a reliable method to evaluate the antibiofilm activity of cements after their initial setting. Studies on the antibiofilm activity of MTA and MTA- and PC-based materials have not been reported. Thus, the aim of this study was to evaluate the antibiofilm activity, compressive strength, and radiopacity of PC associated with ZrO_2_ and nanoparticulated ZnO at different proportions (5% and 10%).

## 2. Materials and Methods 

The evaluated radiopacifiers and the proportions used in their manipulation are described in Table 1. Since this is a preliminary study, Portland cement was used as the main component due its similar composition to commercial MTA.

### 2.1. Antibiofilm Activity

Bovine central incisors with completely formed roots were used as substrate for biofilm development. The roots were sectioned longitudinally. Sections of dentin blocks measuring 5 × 5 × 0.7 mm (width × length × thickness) were obtained by using a diamond disc (Isomet, Buehler, Lake Bluff, IL, USA) at low speed and under irrigation. The resulting blocks were placed in a test tube containing distilled water and sterilized by autoclaving at 121°C for 20 min.

The microbiological procedures and manipulation of the sterilized dentin blocks were carried out in a laminar flow chamber (Veco Flow Ltd., Campinas, SP, Brazil). A standard strain of* Enterococcus faecalis* (ATCC 29212) was used for biofilm formation. After the purity of the strain was confirmed by Gram staining and colony morphology, the microorganism was reactivated in 4.0 mL of sterile brain heart infusion broth (BHI, Difco Laboratories Inc., Detroit, MI, USA) and kept in an oven at 37°C, for 12 hours. The optical density of the medium was measured with a spectrophotometer (Model 600 Plus, Femto, São Paulo, SP, Brazil) set at 600 nm wavelength. Next, an aliquot equivalent to 1% of the volume used for the contamination of the specimens was added and homogenized to the sterile culture medium.

The bovine dentin blocks were placed in 24-well cell culture plates and each well received 2.0 mL of pure BHI broth containing 1% of the bacterial suspension, leaving the dentin blocks all submersed. The plates were kept in an incubator (model Q816M20, Quimis Aparelhos Científicos Ltd., Diadema, SP, Brazil) under constant agitation, in microaerophilic environment at 37°C for 14 days. The BHI medium of each specimen was completely changed every 48 h, without adding new microorganisms to assure enough nutrients for the bacterial cells.

After the postmanipulation periods of 2 days, each material sample was removed from the mould, sterilized through UV light, and positioned over one of the dentin blocks containing biofilm, which were previously washed in saline solution to remove planktonic bacteria. The dentin block/material sample assemblies were placed on 48-well plates. Over each assembly, 20 *µ*L of sterile saline solution was dropped to avoid their drying. The plates were kept in an oven at 37°C for contact times of 5 h and 15 h. Six specimens (dentin block/material) were used for each contact period. The control group comprised noncontact of the biofilm to any cement, allowing the comparison of the results. After the contact periods elapsed, the cement discs were removed and the dentin blocks containing the remaining biofilm, including those belonging to the control group, were individually stored in test tubes containing 1 mL of sterile saline solution and glass pearls. The tubes were agitated in vortex mixer (model Q220, Quimis Aparelhos Científicos Ltd., Diadema, SP, Brazil) for 1 min to disrupt the biofilm.

Following that, serial decimal dilutions of* E. faecalis* were obtained. At the end of the dilutions, three aliquots of 20 *µ*L of each suspension were inoculated onto Petri dishes containing* m-Enterococcus* agar medium (Difco Laboratories, Becton, Dickinson and Company, USA). Dishes were incubated at 37°C, in microaerophilic environment, for 48 h.

The readings for each Petri dish resulted from mean CFU mL^−1^ in the three areas of the bacterial growth, at dilutions that generated between 5 and 50 colonies per field. From these means, the number of CFU mL^−1^ was calculated for each contact period of the filling cements with the biofilm developed onto the dentin blocks. Data were subjected to logarithmic transformation and the result was presented as the mean of the six specimens in each group.

### 2.2. Compressive Strength

The compressive strength was determined according to the method recommended by the BSI [27]. To the test, 6 specimens, measuring 12 mm in height by 6 mm in diameter, were performed as previously described [14, 28]. The specimens were maintained at 37°C and 100% relative humidity by a gauze moistened in distilled water until the tests were performed. Each experimental group was subjected to testing at 24 hours and at 21 days after manipulation of the cements [28].

The surface of each specimen was smoothed using a 600-grit sandpaper. The compression strength was evaluated using a universal testing machine (EMIC DL 2000, Emic Equipamentos, São José dos Pinhais, PR, Brazil) at crosshead speed of 0.5 mm/s with a load of 5 kN. All measurements were recorded in kg and converted to megapascal (MPa).

### 2.3. Radiopacity

The specimens used for radiopacity test were prepared according to the ISO 6876/200121 standard for dental root-sealing materials [29]. Six samples, with 10 mm diameter by 1.0 mm thickness, of each group were subjected to the radiopacity test [14, 25]. After the mixing of the materials, they were stored in 100% humidity at 37°C for 48 hours to set. Subsequently, they were positioned on five occlusal radiographic films (Insight-Kodak Comp, Rochester, NY, USA) and exposed, along with an aluminum stepwedge with variable thickness (from 2 to 16 mm, in 2-mm increments). An X-ray device set at 60 kV, 7 mA, 15 pulses/s, and focal distance of 30 cm was used. Radiographs were digitized using a desktop scanner (SnapScan 1236-Agfa, Deutschland) and the digitized images were imported to the Image Tool 3.0 (UTHSCSA, San Antonio, Texas, USA). The equal-density tool was used to identify equal-density areas in the images [11]. This procedure allowed comparison between the radiographic density of the cements and the radiopacity of the different aluminum stepwedge thicknesses. The area corresponding to the specimen was selected in each radiographic image to verify the thickness of the aluminum stepwedge detected by the software as equivalent to the material's radiographic density. Thus, the radiopacity of the evaluated materials was estimated from the thickness of aluminum (in millimeters) by using a conversion equation [11]. The values recorded for each material were averaged to obtain a single value in mmAl.

### 2.4. Statistical Analysis

The data were subjected to normality test. Once the normal distribution was verified, data were submitted to ANOVA (parametric test) and Tukey tests (*P* ≤ 0.05).

## 3. Results

### 3.1. Antibiofilm Activity

All experimental groups showed similar antibiofilm activity which was greater than control group (*P* < 0.05). The biofilm was not completely removed in any of the evaluated groups (Table 2).

### 3.2. Compressive Strength

At both periods, assessed, pure PC obtained compression strength higher than the other materials. The addition of 5% and 10% nanoparticulated ZnO decreased the compression strength at both experimental periods (Table 2).

### 3.3. Radiopacity

The addition of different radiopacifiers significantly increased the radiopacity of the materials when compared to pure PC. PC + ZrO_2_ + ZnO (10%) exhibited radiopacity higher than other groups (Table 2).

## 4. Discussion

The agar diffusion antimicrobial test allows the comparison of the antimicrobial effect of different materials, but it has limitations once its results depend directly on the material solubility. Other methods to assess antimicrobial activity use the direct contact of the materials to planktonic bacteria [30].

Notwithstanding, the microorganisms of the root canal system are organized as biofilm, which makes them more resistant. In the present study, the methodology enabled the antimicrobial effect evaluation of cements after setting on bacteria organized as biofilm, according to Faria-Júnior et al. [26], but different from the studies on materials over planktonic bacteria.* Enterococcus faecalis* is capable of surviving in alkaline environments and forming biofilm [31]. The bovine dentin substrate provides adequate biofilm formation after the period of 14 days [32].

The use of nanoparticulated ZnO was evaluated by Seil and Webster [33], demonstrating its antimicrobial effect against suspensions of* S. aureus* and* P. aeruginosa* [24]. Kishen et al. [21] showed ZnO antimicrobial effect against* E. faecalis *suspension. Shrestha et al. [22] evaluated ZnO both on biofilm and on planktonic* E. faecalis*, showing bacteria elimination in suspension and the decrease of biofilm thickness, which is in agreement with the results of this study, as observed for all materials.

Regarding MTA- and PC-based materials, their antimicrobial effect was demonstrated by Asgary and Kamrani [34] using agar diffusion test. Guerreiro-Tanomaru et al. [32] showed antimicrobial effectiveness of PC associated with different radiopacifiers, including ZrO_2_.

Although ZnO has presented a satisfactory antimicrobial effect in other researches [21–23, 33], in the present study, the materials with ZnO showed partial effect on* E faecalis* biofilm. This result can be associated with the hydration reaction of MTA with ZnO particles, resulting in a material with low solubility and, consequently, reducing the antimicrobial effect.

The reduction of bacteria observed in the other groups can be related to high pH values exhibited by PC-based materials. According to Guerreiro-Tanomaru et al. [32], the pH of either pure PC or PC in addition to different radiopacifiers including ZrO_2_ is about 10.2. Faria-Júnior et al. [26] observed similar results for MTA, with values close to the required to achieve effectiveness against* E. faecalis *[35].

The results of the present study demonstrated that PC associated with microparticulated ZrO_2_ and nanoparticulated ZnO present an adequate radiopacity, ranging from 3.50 mmAl to 3.92 mmAl, above the minimum value recommended by ANSI/ADA specification n.57 of 3 millimeters of aluminum [36]. These findings are in accordance with those values previously observed for PC associated with ZrO_2_ by Bortoluzzi et al. [10].

Regarding compression strength, all materials showed higher values at 21-day period when compared with 24-hour period. This increase in resistance occurs because of the ending of hydration phase, providing more hardness to the material. Saldana et al. [37] demonstrated that ZrO_2_ favors the mechanical properties of the material, corroborating the results observed in the present study. Notwithstanding, the values of the association PC + ZrO_2_ were smaller than PC which is in accordance with Silva et al. [14].

Furthermore, the nanoparticulated zinc oxide dramatically reduced the compressive strength of the materials, especially at 24 hours. At least in part, this finding can be related to the reaction mechanism of nanoparticles with PC and ZrO_2_, culminating in a material with flaws in the microstructure and low compressive strength.

ZrO_2_ seems to be an adequate alternative radiopacifier for PC since it shows biocompatibility and low toxicity [14], provides satisfactory radiopacity [10, 14] and physicochemical properties [14, 15, 17, 18], and maintains the antimicrobial activity of PC [32]. However, considering the results of the present study, further researches are needed for better comprehension of the interaction between ZnO nanoparticles and calcium silicate-based cements with ZrO_2_.

MTA was formulated from commercial Portland cement combined with bismuth oxide powder for radiopacity. However, the composition, fineness, setting time, and strength of Portland cement are not controlled or guaranteed. Portland cement is an unsuitable substitute for MTA based on several characteristics that are essential to the MTA performance. Thus, this study was performed in order to evaluate the effect of the association of zinc oxide nanoparticles in physicochemical and antimicrobial properties of calcium silicate cements. Additional studies are required for this or similar associations in order to verify the possibilities for clinical use.

## 5. Conclusion

It can be concluded that the addition of ZrO_2_ and ZnO does not interfere in the antibiofilm activity and provides radiopacity to Portland cement. However, the presence of ZnO significantly decreased the compressive strength of the material.

## Figures and Tables

**Table 1:** Experimental materials and their manufacturers.

Group	Material	Manufacturer
PC	Portland cement (PC) 330 *µ*L of distilled water	PC-CPB-40 structural Votoran, Votorantim Cimentos, Brazil

PC + ZrO_2_	PC (70%) + ZrO_2_ (30%) 330 *µ*L of distilled water	PC-CPB-40 structural Votoran, Votorantim Cimentos, Brazil ZrO_2_—Sigma-Aldrich Brazil Ltd., São Paulo, SP, Brazil

PC + ZrO_2_ + ZnO (5%)	PC (65%) + ZrO_2_ (30%) + ZnO (5%) 330 *µ*L of distilled water	PC-CPB-40 structural Votoran, Votorantim Cimentos, Brazil ZrO_2_—Sigma-Aldrich Brazil Ltd., São Paulo, SP, Brazil ZnO—Nanotechnology Laboratory of the Physics Institute of São Carlos, SP, Brazil

PC + ZrO_2_ + ZnO (10%)	PC (60%) + ZrO_2_ (30%) + ZnO (10%) 330 *µ*L of distilled water	PC-CPB-40 structural Votoran, Votorantim Cimentos, Brazil ZrO_2_—Sigma-Aldrich Brazil Ltd., São Paulo, SP, Brazil ZnO—Nanotechnology Laboratory of the Physics Institute of São Carlos, SP, Brazil

PC: Portland cement.

ZrO_2_: zirconium oxide.

ZnO: zinc oxide.

**Table 2:** Mean and standard deviation values of the different cements tested.

Tests	PC	PC + ZrO_2_	PC + ZrO_2_ + ZnO (5%)	PC + ZrO_2_ + ZnO (10%)	Control
DCT-5 h (UFC)	5.84^a^ ± 0.44	5.67^a^ ± 0.67	5.90^a^ ± 0.40	5.72^a^ ± 0.36	7.11^b^ ± 0.48
DCT-15 h (UFC)	5.07^a^ ± 3.78	5.09^a^ ± 0.15	5.06^a^ ± 0.67	5.15^a^ ± 0.46	6.85^b^ ± 0.18

24 h compression strength (MPa)	32.11^a^ ± 3.64	24.09^b^ ± 1.22	0.58^c^ ± 0.03	0.62^c^ ± 0.06	—
21 d compression strength (MPa)	67.88^a^ ± 7.35	57.43^b^ ± 7.0	30.90^c^ ± 5.32	27.83^c^ ± 4.77	—

Radiopacity (mmAl)	1.15^c^ ± 0.12	3.70^b^ ± 0.06	3.50^b^ ± 0.16	3.92^a^ ± 0.18	—

Mean ± standard deviation.

Similar letters indicate statistical similarity (*P*≥ 0.05).
